# Relationship between biologic therapy and cytokine levels in patients with inflammatory arthritis

**DOI:** 10.1097/MD.0000000000042953

**Published:** 2025-06-20

**Authors:** Ze Zhang, Bing-He Zhou, Liang Hu, Ming-Chao Li, Dong Chang, Xin Dou

**Affiliations:** a Department of Clinical Laboratory, Shanghai Pudong Hospital, Fudan University, Shanghai, China.

**Keywords:** ankylosing spondylitis, biologics, cytokines, inflammatory arthritis, rheumatoid arthritis

## Abstract

Biological agents are frontline treatments for ankylosing spondylitis (AS) and rheumatoid arthritis (RA); however, their efficacy varies owing to differences in patient autoimmune status. This study aimed to evaluate peripheral blood cytokine profiles in patients with AS and RA and assess the impact of different treatment modalities. Data from 145 patients with AS, 491 patients with RA, and 125 healthy controls were collected. Cytokine levels (tumor necrosis factor [TNF]-α, interleukin [IL]-6, IL-8, IL-17, IL-5, etc) were analyzed in healthy controls and patients with AS and RA, with a focus on patients with AS treated with or without biologics. The effects of biologic therapy on cytokine levels in patients with AS and RA were evaluated, along with the impact of disease-modifying antirheumatic drug (DMARD) monotherapy or combination therapy with biologics in patients with RA. Significant differences in interferon (IFN)-α, IFN-γ, IL-10, IL-12P70, IL-2, IL-4, IL-5, IL-6, IL-8, TNF-α, and IL-1β were found among the AS, RA, and HC groups (*P* < .001); however, no significant difference was noted in IL-17A levels. In patients with RA treated with biologics, IL-1β levels significantly increased (*P* = .013). In patients with AS treated with biologics, TNF-α levels were correlated with IL-6 and IL-8 levels, and IL-17A levels were correlated with IL-5 and IL-8 levels. Combination therapy with DMARDs and biologics significantly increased IL-1β (*P* = .016) and IL-6 (*P* = .047) levels in patients with RA. Overall, biological agents affect cytokine levels in patients with AS and RA, and combination therapy with DMARDs upregulates these levels. Drug choice and combination may significantly influence treatment outcomes in patients with AS and RA treated with biological agents.

## 1. Introduction

Inflammatory arthritis is a chronic inflammatory disease characterized by persistent joint erosion, cartilage destruction, and synovial hyperplasia.^[1]^ Ankylosing spondylitis (AS) and rheumatoid arthritis (RA) are common manifestations of inflammatory arthritis. The clinical manifestations and pathogenic mechanisms of AS and RA vary across different studies.^[2,3]^ RA primarily affects peripheral joints, with synovial inflammation and articular destruction mainly driven by pro-inflammatory cytokines such as tumor necrosis factor (TNF-α), which promotes synoviocyte proliferation, inflammatory mediator release, and osteoclast activation, and IL-6, which induces acute-phase reactants (e.g., C-reactive protein [CRP]), B-cell activation, and osteoclastogenesis. These cytokines form a vicious cycle through autocrine and paracrine pathways, continuously amplifying inflammation and ultimately leading to joint structural damage.^[4]^ AS mainly involves the spine and is manifested as sacroiliitis and spinal involvement. AS pathogenesis is closely associated with excessive activation of inflammatory factors such as TNF-α and the interleukin (IL)-23/IL-17 axis. These factors drive articular inflammation and contribute to extra-articular manifestations such as uveitis.^[5]^ Long-term chronic inflammation and immune dysregulation are common features of both RA and AS.^[6]^

Cytokines secreted by immune cells, such as T, B, and NK cells, are key mediators of the immune response and have an important impact on the pathophysiology of both diseases.^[7]^ In recent years, the clinical treatment of RA and AS has changed from non-pharmacological anti-inflammatory drugs to synthetic antirheumatic medicines and subsequently to various biological drugs targeting inflammatory cytokines, providing a variety of treatment options for most patients.^[8,9]^ Studies have shown that high levels of TNF-α are closely related to the disease development and inflammatory process of RA and AS.^[10]^ TNF-α, a pro-inflammatory cytokine, can aggravate the inflammatory response and bone and joint destruction.^[11]^ Currently, TNF-α inhibitors have been used in clinical practice; for instance, adalimumab, a monoclonal antibody biological agent, is widely used in the treatment of AS, RA, and other related autoimmune diseases and acts by neutralizing or blocking the activity of TNF-α.^[12,13]^ Secukinumab is a biological agent that targets IL-17A. Studies have reported that it can significantly relieve back pain and disease activity in patients with AS, reduce joint swelling and tenderness in patients with RA, and improve the quality of life of patients.^[14,15]^

Although biological agents have significant effects on AS and RA, they present safety concerns.^[16]^ In addition to various biological agents, it is important to consider individual treatments for patients. Changes in cytokine levels reflect the degree of inflammation and can be used to monitor treatment effects.^[17]^ Therefore, in this study, we first analyzed the changes in peripheral blood cytokine profiles in healthy controls (HCs) as well as patients with AS and RA and then divided the participants into further groups according to the use of disease-modifying antirheumatic drug (DMARDs) and biological agents to determine the cytokines that play a major role in the treatment of patients with AS and RA.

## 2. Methods

### 2.1. Patients

From January 2023 to April 2024, 761 patients with rheumatic immune diseases were recruited from Pudong Hospital, Affiliated with Fudan University in the People’s Republic of China, to participate in our study. These included 125 HCs, 145 patients with AS, and 491 patients with RA. Clinical data of the patients, including age, sex, and treatment with steroids, nonsteroidal anti-inflammatory drugs (NSAIDs), DMARDs (such as sulfasalazine, methotrexate, and leflunomide), and biological agents (adalimumab and secukinumab) alone or in combination, were collected from electronic medical records. Laboratory indicators included peripheral blood cytokines, human leukocyte antigen (HLA)-B27, erythrocyte sedimentation rate (ESR), CRP, rheumatoid factor (RF), anti-RF-IgM, anti-RF-IgG, anti-RF-IgA, and anti-cyclic citrullinated peptide (CCP) levels. The following cases were included: those that met the 2010 American College of Rheumatology and European Society of Rheumatology diagnostic criteria for RA and the modified New York diagnostic criteria for AS, those wherein the patient age was 18 years or above, and those that have complete general patient information. Exclusion criteria included malignant tumors, severe infection, heart failure, severe hepatic and renal insufficiency, and pregnancy. This study was conducted in adherence to the principles of the Helsinki Declaration (2013) and the ethical guidelines outlined in China’s Ethical Review Measures for Biomedical Research Involving Humans. Given that this is a retrospective study utilizing de-identified clinical data collected from an electronic medical record system, the Ethics Committee of Pudong Hospital affiliated with Fudan University (approval No.: 2024-IIT-013-E01) granted a waiver of individual informed consent.

### 2.2. Peripheral blood cytokine assay

The multiplex cytokine bead array technology was employed using the Cytokine Multiplex Detection Kit (Jiangxi Cellgene Biotech Co., Ltd. No. P110100403) to simultaneously measure 12 cytokines, including IL-1β, IL-2, IL-4, IL-5, IL-6, IL-8, IL-10, IL-12p70, IL-17A, TNF-α, interferon (IFN)-γ, and IFN-α. The detailed procedure was as follows. Venous blood samples were collected in EDTA-coated tubes and centrifuged at 1500 *g* for 10 minutes. Approximately 0.025 mL of separated plasma was aliquoted for testing. A 0.025-mL volume of mixed microsphere capture solution and 0.025 mL of fluorescent antibody were then added, followed by incubation at 25 ℃ in the dark for 2.5 hours. Each tube was then washed with 1 mL phosphate buffered saline solution and centrifuged at 200 *g* for 5 minutes; supernatants were then carefully aspirated. Finally, 100 μL of phosphate buffered saline solution was added to each tube for resuspension, and samples were stored in the dark pending analysis. Data acquisition was performed using BD FACS Diva software on a BD FACS Canto II flow cytometer (BD Biosciences, USA), with subsequent analysis conducted via FCAP Array software.

### 2.3. Statistical analysis

SPSS (version 27.0, USA) and GraphPad Prism (version 9.0, USA) were used for statistical analyses. Count data were expressed as n (%) and were analyzed using the chi-square test. Measurement data that were not normally distributed were expressed as medians (P25, P75) and analyzed using non-parametric tests. Comparisons between groups were performed using the Mann–Whitney (*U* test) or Kruskal–Wallis test (*H* test) and Bonferroni multiple comparison test. Pearson correlation analysis was used to analyze the correlation between cytokines in patients with AS or RA treated with different drugs. A *P*-value < .05 was considered statistically significant.

## 3. Results

### 3.1. Comparison of clinical characteristics of HCs and patients with AS and RA

The clinical characteristics and laboratory parameters of HCs and patients with RA and AS are shown in Table 1. The median age of patients with RA was significantly higher than that of patients with AS (*P* < .001). Furthermore, 56.6% of patients with AS were male; this proportion was significantly higher than that among patients with RA (26.1%; *P* < .001). HLA-B27 was positive in 86.2% of patients with AS. There was no significant difference in CRP and anti-RF-IgG levels between patients with RA and AS; however, ESR, RF, anti-RF-IgM, anti-RF-IgA, and anti-CCP levels were higher in patients with RA than in patients with AS (*P* < .001).

**Table 1:** Baseline clinical characteristics of healthy controls, patients with ankylosing spondylitis, and patients with rheumatoid arthritis.

Characteristics	HC (n = 125)	AS (n = 145)	RA (n = 491)	*P*-value
Male, sex	60 (48.00)	82 (56.60)	128 (26.10)	<.001
Age, years	44 (36, 52.5)	48 (35, 60)	64 (54, 70)	<.001
HLA-B27 positive	–	125 (86.20)	–	–
ESR, mm/h	–	9 (4, 20)	15 (7, 31)	<.001
CRP, mg/L	–	2.52 (0.50, 11.81)	1.74 (0.50, 8.89)	.504
RF, Iu/mL	–	6.87 (3.64, 18.89)	42.77 (10.45, 117.28)	<.001
Anti-RF-IgM, Iu/mL	–	13.11 (4.27, 35.15)	61.04 (13.00, 244.96)	<.001
Anti-RF-IgG, Iu/mL	–	3.36 (2.00, 5.73)	3.77 (2.46, 7.17)	.058
Anti-RF-IgA, Iu/mL	–	6.60 (4.54, 10.16)	12.93 (6.77, 36.43)	<.001
Anti-CCP, Iu/mL	–	2.63 (2.00, 14.21)	36.78 (2.39, 400.00)	<.001
Treated with steroid	–	56 (38.62)	320 (65.17)	<.001
Treated with NSAIDs	–	106 (73.10)	339 (69.04)	.349
Treated with DMARDs	–	99 (68.28)	470 (95.72)	<.001
Treated with biologics	–	117 (80.69)	162 (32.99)	<.001
Adalimumab	–	96 (66.21)	150 (30.55)	–
Secukinumab	–	21 (14.48)	12 (2.44)	–

AS = ankylosing spondylitis, CCP = cyclic citrullinated peptide, CRP = C-reactive protein, DMARD = disease-modifying antirheumatic drug, ESR = erythrocyte sedimentation rate, HC = healthy controls, HLA = human leukocyte antigen, NSAIDs = nonsteroidal anti-inflammatory drugs, RA = rheumatoid arthritis, RF = rheumatoid factor.

There were some differences in the current first-line clinical drug treatment between the RA and AS groups; however, there were no significant differences in the use of NSAIDs between the 2 groups. Compared with patients with AS, those with RA had a higher proportion of treatments with steroids and DMARD (*P* < .001), and patients with AS had a higher proportion of treatments with biologics (*P* < .001). The main biological drugs used in patients with AS and patients with RA were adalimumab and secukinumab, respectively.

### 3.2. Levels of peripheral blood cytokines in HCs and patients with AS and RA

**Figure 1.**
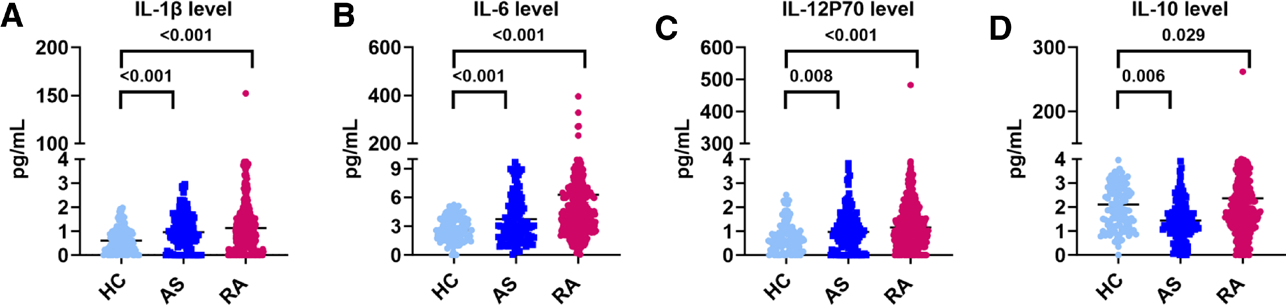
Cytokine levels in HCs and patients with AS and RA. (A) IL-1β, (B) IL-6, (C) IL-12P70, (D) IL-10. (HC, n = 125, AS, n = 145, RA, n = 491). AS = ankylosing spondylitis, HC = healthy controls, IL = interleukin, RA = rheumatoid arthritis.

The levels of the 12 cytokines in the peripheral blood of the AS and RA groups are shown in Table 2. The levels of IFN-α, IFN-γ, IL-10, IL-12P70, IL-1β, IL-2, IL-4, IL-5, IL-6, IL-8, and TNF-α in patients with RA were significantly higher than those in patients with AS and HC (*P* < .001). There was no significant difference in IL-17A levels among the 3 groups. We further conducted a multi-comparison between groups for the cytokines with significant differences, and the expression levels of IL-1β, IL-6, IL-12P70, and IL-10 were significantly higher in the AS and RA groups than in the HC group (Fig. 1A–D).

**Table 2:** Cytokine levels in healthy controls, patients with ankylosing spondylitis, and patients with rheumatoid arthritis.

Characteristics	HC (n = 125)	AS (n = 145)	RA (n = 491)	*P*-value
IFN-α, pg/mL	0.75 (0.35, 1.34)	0.81 (0.47, 1.40)	1.09 (0.61, 2.44)	<.001
IFN-γ, pg/mL	1.38 (0.67, 2.32)	1.22 (0.41, 1.97)	1.74 (0.89, 2.80)	<.001
IL-10, pg/mL	2.11 (1.26, 2.95)	1.44 (0.99, 2.29)	2.36 (1.43, 4.14)	<.001
IL-12P70, pg/mL	0.60 (0.39, 1.02)	0.97 (0.45, 1.53)	1.16 (0.61, 2.08)	<.001
IL-17A, pg/mL	0.78 (0.33, 2.40)	1.49 (0.00, 6.86)	1.38 (0.13, 4.91)	.079
IL-1β, pg/mL	0.60 (0.24, 1.00)	0.96 (0.53, 1.68)	1.13 (0.50, 2.19)	<.001
IL-2, pg/mL	0.92 (0.32, 1.53)	0.87 (0.46, 1.34)	1.39 (0.68, 2.98)	<.001
IL-4, pg/mL	0.88 (0.47, 1.75)	0.83 (0.23, 1.70)	1.51 (0.63, 2.93)	<.001
IL-5, pg/mL	0.41 (0.18, 0.68)	0.46 (0.25, 0.74)	0.63 (0.34, 1.16)	<.001
IL-6, pg/mL	2.58 (1.74, 3.70)	3.73 (1.95, 8.12)	6.28 (3.35, 13.53)	<.001
IL-8, pg/mL	7.14 (4.51, 10.6)	6.84 (4.64, 9.17)	9.52 (6.74, 14.75)	<.001
TNF-α, pg/mL	0.97 (0.54, 1.54)	0.97 (0.39, 1.89)	1.37 (0.61, 2.49)	<.001

AS = ankylosing spondylitis, HC = healthy controls, IFN = interferon, IL = interleukin, RA = rheumatoid arthritis, TNF = tumor necrosis factor.

### 3.3. Levels of peripheral blood cytokines between non-biologics and biologics groups of patients with AS and RA

**Figure 2.**
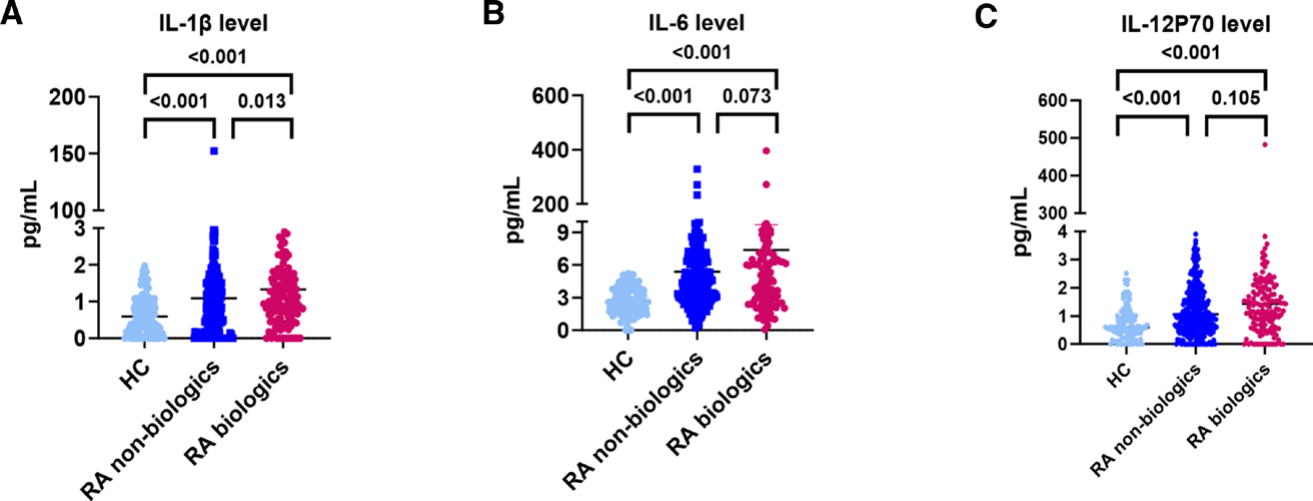
Differences in cytokine levels between the RA group with non-biologics and that with biologics. (A) IL-1β, (B) IL-6, (C) IL-12P70. (HC, n = 125; RA, non-biologics, n = 329, biologics, n = 162). HC = healthy controls, IL = interleukin, RA = rheumatoid arthritis.

The patients with AS and RA were divided into 4 groups according to whether biologics were used, and the changes in peripheral blood cytokine levels were compared (Table 3). There were differences in IL-10, IL-1β, and IL-6 among HC and patients with AS with or without biologics; however, further multiple comparisons were not significant. The levels of IFN-α, IFN-γ, IL-10, IL-12P70, IL-1β, IL-2, IL-4, IL-5, IL-6, IL-8, and TNF-α differed among patients with RA treated with biologics (*P* < .001, *P* = .006, *P* = .013 respectively). Further multiple comparisons showed that the IL-1β level was significantly increased in the RA group with biologics (Fig. 2A). IL-6 and IL-12P70 showed an increasing trend in the group using biologics for RA (Fig. 2B and C).

**Table 3:** Peripheral blood cytokine levels in patients with ankylosing spondylitis and rheumatoid arthritis with and without biologics.

Characteristics	HC	AS		*P*-value	HC	RA		*P*-value
	(n = 125)	Non-biologics (n = 28)	Biologics (n = 117)		(n = 125)	Non-biologics (n = 329)	Biologics (n = 162)	
IFN-α, pg/mL	0.75 (0.35, 1.34)	0.80 (0.37, 1.25)	0.83 (0.50, 1.44)	.313	0.75 (0.35, 1.34)	1.07 (0.61, 2.31)	1.13 (0.66, 2.70)	<.001
IFN-γ, pg/mL	1.38 (0.67, 2.32)	1.19 (0.65, 2.05)	1.24 (0.28, 1.97)	.166	1.38 (0.67, 2.32)	1.83 (1.04, 2.85)	1.57 (0.70, 2.77)	.013
IL-10, pg/mL	2.11 (1.26, 2.95)	1.18 (0.85, 1.98)	1.49 (1.02, 2.33)	<.001	2.11 (1.26, 2.95)	2.48 (1.45, 4.31)	2.13 (1.35, 4.07)	.006
IL-12P70, pg/mL	0.60 (0.39, 1.02)	0.99 (0.48, 1.55)	0.97 (0.45, 1.53)	.006	0.60 (0.39, 1.02)	1.06 (0.57, 1.98)	1.43 (0.74, 2.26)	<.001
IL-17A, pg/mL	0.78 (0.33, 2.40)	2.05 (0.00, 7.86)	1.27 (0.00, 6.54)	.416	0.78 (0.33, 2.40)	1.37 (0.34, 4.36)	1.52 (0.00, 5.57)	.058
IL-1β, pg/mL	0.60 (0.24, 1.00)	1.07 (0.53, 1.56)	0.94 (0.49, 1.68)	<.001	0.60 (0.24, 1.00)	1.08 (0.43, 1.90)	1.33 (0.74, 2.64)	<.001
IL-2, pg/mL	0.92 (0.32, 1.53)	0.95 (0.32, 1.33)	0.84 (0.46, 1.37)	.989	0.92 (0.32, 1.53)	1.45 (0.68, 2.96)	1.39 (0.68, 3.12)	<.001
IL-4, pg/mL	0.88 (0.47, 1.75)	0.84 (0.32, 1.91)	0.77 (0.21, 1.66)	.79	0.88 (0.47, 1.75)	1.57 (0.66, 2.97)	1.44 (0.54, 2.85)	<.001
IL-5, pg/mL	0.41 (0.18, 0.68)	0.46 (0.29, 0.82)	0.47 (0.24, 0.74)	.339	0.41 (0.18, 0.68)	0.61 (0.33, 1.16)	0.63 (0.36, 1.13)	<.001
IL-6, pg/mL	2.58 (1.74, 3.70)	3.50 (2.02, 5.76)	3.75 (1.91, 8.53)	<.001	2.58 (1.74, 3.70)	5.37 (3.30, 11.18)	7.40 (3.56,17.97)	<.001
IL-8, pg/mL	7.14 (4.51, 10.6)	7.47 (5.58, 8.89)	6.84 (4.46, 9.22)	.717	7.14 (4.51, 10.6)	9.71 (6.84, 15.09)	9.17 (6.42, 14.19)	<.001
TNF-α, pg/mL	0.97 (0.54, 1.54)	0.84 (0.28, 2.22)	0.97 (0.41, 1.82)	.899	0.97 (0.54, 1.54)	1.42 (0.62, 2.54)	1.34 (0.60, 2.43)	<.001

AS = ankylosing spondylitis, HC = healthy controls, IFN = interferon, IL = interleukin, RA = rheumatoid arthritis, TNF = tumor necrosis factor.

### 3.4. Correlation between peripheral blood cytokine levels and use of biologics in the AS and RA group

**Figure 3.**
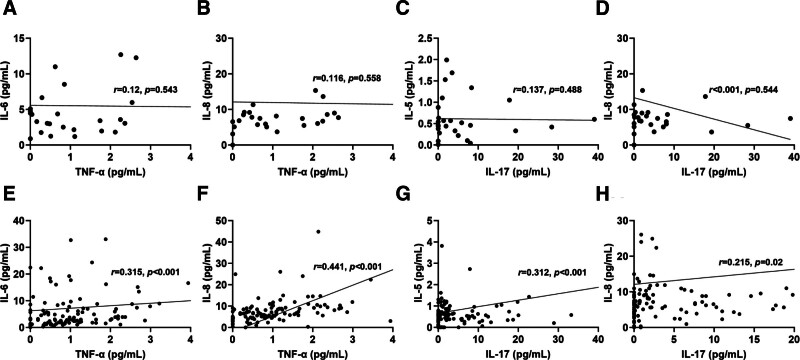
Correlation between cytokine levels in the AS group with non-biologics and that with biologics. (A–D) IL-6 and TNF-α, IL-8 and TNF-α, IL-5 and IL-17A, and IL-8 and IL-17A (AS, non-biologics, n = 28). (E–H) IL-6 and TNF-α, IL-8 and TNF-α, IL-5 and IL-17A, IL-8 and IL-17A (AS, biologics, n = 117). AS = ankylosing spondylitis, IL = interleukin, TNF = tumor necrosis factor.

Subsequently, we analyzed the correlation between cytokine levels in patients with AS treated with and without biologics. We found no correlation between IL-6 and TNF-α (*R* = 0.12), IL-8 and TNF-α (*R* = 0.116), IL-5 and IL-17A (*R* = 0.137), and IL-8 and IL-17A (*R* < 0.001) in patients with AS who were not treated with biologics (Fig. 3A–D); however, in patients with AS who were treated with biologics, IL-6 and IL-8 levels increased with levels of TNF-α (*R* = 0.315 and *R* = 0.441, respectively) (Fig. 3E, F), and IL-5 and IL-8 levels increased with levels of IL-17A (*R* = 0.312 and *R* = 0.215, respectively) (Fig. 3G, H). These results demonstrate a significant correlation of IL-6 and IL-8 with TNF-α and of IL-5 and IL-8 with IL-17A in peripheral blood of patients with AS after treatment with biologic agents. We also performed correlation analyses between IL-6 and ESR/CRP in patients with RA treated with non-biologic agents versus biologic agents to evaluate disease severity. Positive correlations were observed between IL-6 and ESR/CRP in the non-biologics group (*R* = 0.334 and *R* = 0.279, respectively) (Figure S1A, B, Supplemental Digital Content, https://links.lww.com/MD/P233). Similarly, significant positive correlations were detected in the biologics group (*R* = 0.375 and *R* = 0.333, respectively) (Figure S1C, D, Supplemental Digital Content, https://links.lww.com/MD/P233).

### 3.5. Effect of DMARDs or biologics on cytokine levels in patients with RA

**Figure 4.**
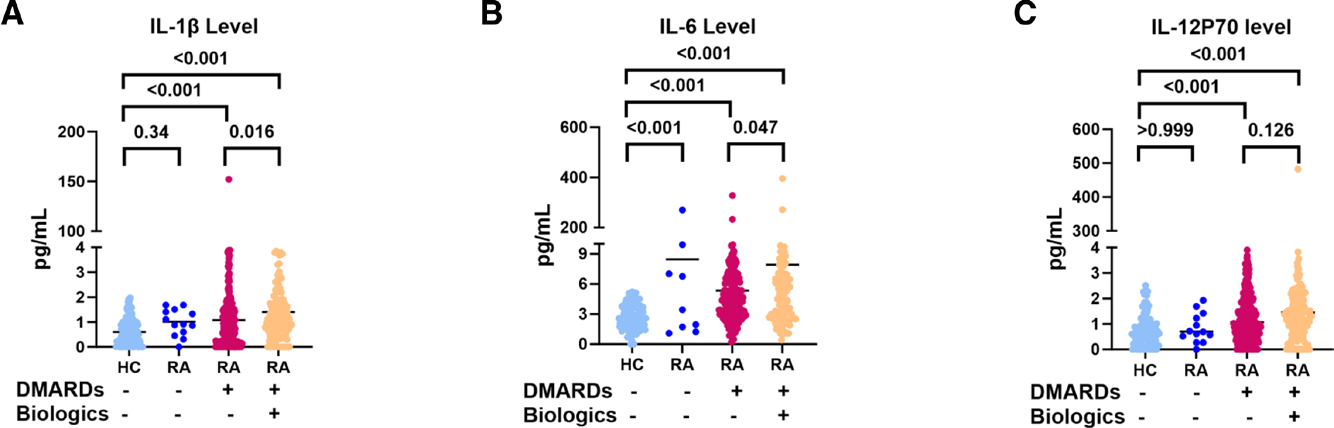
Differences in (A) IL-1β, (B) IL-6, and (C) IL-12P70 levels in HCs (n = 125) and patients with RA in the non-DMARD and non-biologics groups (n = 14), DMARD-only group (n = 315), and combined DMARD and biologics group (n = 155). DMARD = disease-modifying antirheumatic drug, HC = healthy controls, IL = interleukin, RA = rheumatoid arthritis.

Next, we compared cytokine levels in patients with RA treated with a combination of DMARDs and biologics (Table 4). According to the medication information of patients with RA, the patients were divided into 3 groups: no use of DMARDs and biologics, use of DMARDs, and combined use of DMARDs and biologics. All cytokine levels were significantly different among the 4 groups (*P* < .001, *P* = .009, *P* = .04, *P* = .042 respectively). Subsequently, we performed multiple comparisons of the differentially expressed cytokines. IL-1β and IL-6 levels were significantly increased in the combined DMARDs and biologics groups (Fig. 4A, B). There were no significant differences in IL-12P70 levels among the 4 groups (Fig. 3C). We also analyzed the levels of cytokine changes in patients with AS treated with DMARDs alone or combined with biologics, and there were significant changes in IL-1β and IL-6 levels; however, no significant differences were noted in multiple comparisons (Table S1, Supplemental Digital Content, https://links.lww.com/MD/P234).

**Table 4:** Effect of treatment with disease-modifying antirheumatic drugs or biologics on different cytokine levels in rheumatoid arthritis.

Characteristics	HC	RA			*P*-value
DMARDs	-	-	+	+	
Biologics	-	-	-	+	
Number of participants	125	14	315	155	
IFN-α, pg/mL	0.75 (0.35, 1.34)	0.73 (0.52, 1.96)	1.07 (0.61, 2.37)	1.15 (0.68, 2.93)	<.001
IFN-γ, pg/mL	1.38 (0.67, 2.32)	1.69 (1.00, 2.92)	1.83 (1.04, 2.85)	1.57 (0.71, 2.78)	.040
IL-10, pg/mL	2.11 (1.26, 2.95)	4.64 (1.34, 7.50)	2.46 (1.45, 4.21)	2.20 (1.41, 4.10)	.009
IL-12P70, pg/mL	0.60 (0.39, 1.02)	0.71 (0.46, 1.49)	1.07 (0.57, 2.02)	1.45 (0.82, 2.29)	<.001
IL-17A, pg/mL	0.78 (0.33, 2.40)	1.14 (0.23, 1.61)	1.38 (0.34, 4.70)	1.65 (0.00, 5.88)	.042
IL-1β, pg/mL	0.60 (0.24, 1.00)	1.01 (0.56, 1.55)	1.08 (0.43, 1.93)	1.40 (0.74, 2.76)	<.001
IL-2, pg/mL	0.92 (0.32, 1.53)	1.06 (0.62, 2.84)	1.46 (0.68, 2.96)	1.44 (0.72, 3.13)	<.001
IL-4, pg/mL	0.88 (0.47, 1.75)	1.24 (0.14, 3.17)	1.58 (0.70, 2.96)	1.45 (0.56, 2.85)	<.001
IL-5, pg/mL	0.41 (0.18, 0.68)	0.59 (0.41, 1.01)	0.63 (0.32, 1.16)	0.63 (0.38, 1.12)	<.001
IL-6, pg/mL	2.58 (1.74, 3.70)	8.46 (1.89, 50.27)	5.35 (3.30, 11.14)	7.92 (3.64, 18.83)	<.001
IL-8, pg/mL	7.14 (4.51, 10.6)	10.21 (7.65, 16.71)	9.68 (6.84, 14.98)	9.21 (6.53, 14.56)	<.001
TNF-α, pg/mL	0.97 (0.54, 1.54)	0.54 (0.14, 1.18)	1.45 (0.66, 2.57)	1.36 (0.67, 2.90)	<.001

DMARD = disease-modifying antirheumatic drug, HC = healthy controls, IFN = interferon, IL = interleukin, RA = rheumatoid arthritis, TNF = tumor necrosis factor.

## 4. Discussion

We retrospectively analyzed the electronic medical records of 491 patients with RA, 145 patients with AS, and 125 HCs. It is important to note that AS is closely related to HLA-B27. In our study, 86.2% of patients with AS tested positive for HLA-B27. RA was characterized by autoantibodies, such as RF, anti-RF-IgM, anti-RF-IgG, and anti-RF-IgA, and the anti-CCP antibody positivity rate in patients with RA was significantly higher than that in patients with AS. These differences in immunological characteristics suggest different underlying pathogenic mechanisms.

We examined different cytokine levels in the peripheral blood of patients with RA and AS and the effects of DMARDs and biologics on cytokine profiles. Our results revealed that IFN-α, IFN-γ, IL-10, IL-12P70, IL-2, IL-4, IL-5, IL-6, IL-8, TNF-α, and IL-1β levels were all significantly increased in patients with RA compared with those in patients with AS. This indicates that an increase in these cytokine levels can trigger synovial joint inflammation and trigger immune cells to produce more cytokines, forming a positive feedback loop and leading to the continuous development of immune dysregulation, thus creating a complex cytokine regulatory network in patients with RA.

T helper (Th) cells are important in regulating the immune response. Th cells, especially Th1, Th2, and Th17 cells, are involved in the development of autoimmune diseases, such as RA and AS, by secreting various cytokines.^[18,19]^ Th1 cells can secrete pro-inflammatory cytokines, such as IFN-γ and TNF-α.^[20]^ Studies have shown that continuous increases in the levels of IFN-γ and TNF-α in patients with RA can promote macrophage invasion, increase inflammatory responses, and lead to joint damage.^[21]^ However, Th2 cells mainly secrete cytokines such as IL-4, IL-5, IL-10, and IL-13, which play a role in humoral immunity.^[22,23]^ Studies have shown that the balance between Th1 and Th2 cells plays an important role in the body. Th1/Th2 imbalance promotes the continuous activation of immune cells, which leads to allergic inflammation, such as asthma, and the development of autoimmune diseases.^[24]^ Th17 cells are another subset of CD4 + T cells that secrete IL-17 and IL-21.^[25]^ Studies have shown that the number of Th17 cells is increased in the peripheral blood and synovial fluid of patients with RA and AS.^[26]^ IL-17 promotes inflammation by stimulating TNF-α production and neutrophil recruitment in RA.^[27]^ We investigated the relationship between cytokines secreted by Th1, Th2, Th17, and other immune cells in the peripheral blood and the pathogenesis of AS and RA. In addition, we observed changes in cytokine profiles using DMARDs and biological agents because these agents, alone or in combination, affect peripheral cytokine levels.

Accumulating evidence indicates that in RA, TNF-α and IL-6 induce osteoclast differentiation via the RANKL-independent pathways. Subsequent activation of osteoclasts leads to the secretion of pro-inflammatory cytokines, including IL-1β, TNF-α, and IL-12p40, which significantly exacerbate articular inflammation and destruction. Clinical data demonstrate positive correlations between osteoclast counts and modified Total Sharp Score and between osteoclast differentiation potential and serum CRP levels, suggesting their utility as biomarkers for disease activity assessment and joint destruction prediction. Targeted inhibition of TNF-α/IL-6 signaling may represent a novel therapeutic strategy for retarding RA progression.^[21]^ Jing et al reported significantly elevated serum levels of IL-6 and TNF-α in patients with AS, which were strongly correlated with disease activity (Bath Ankylosing Spondylitis Disease Activity Index), inflammatory markers (CRP, ESR), and imaging grading of sacroiliac joints. Specifically, serum TNF-α levels were significantly increased in patients with AS and hip joint involvement, whereas IL-6 levels showed marked elevation in patients without hip joint involvement but with sacroiliac joint involvement. These cytokines may serve as biomarkers for auxiliary diagnosis, disease progression assessment, and targeted therapy guidance, thereby improving patient outcomes.^[28]^ Our results showed significant increases in IFN-α, IFN-γ, IL-10, IL-12P70, IL-2, IL-4, IL-5, IL-6, IL-8, TNF-α, and IL-1β levels in patients with RA.

We divided the patients into a non-biologics group if they used steroids, NSAIDs, and DMARDs and a biologics group if they used adalimumab or culizumab. We observed whether the biological agents affected the levels of cytokines. Studies have reported that the TNF-α level in AS was significantly lower than that in RA.^[26]^ The IL-17 and IL-23 levels in AS and those without RA did not change significantly. In our study, there was no change in cytokine levels in patients with AS with or without biological agents, which is consistent with the previous study results. Notably, TNF-α was correlated with IL-6 and IL-8, and IL-17 was correlated with IL-5 and IL-8 in the non-biologics group. Furthermore, multiple cytokines were significantly affected after using biologics in patients with RA, including IL-1β, IL-6, and IL-12P70. Biologics may enhance inflammation in patients with RA and increase the risk of infection. In the present study, no significant changes in IL-17A levels were observed. Specifically, 14.48% of patients with AS received secukinumab (an IL-17A inhibitor), compared with only 2.44% of patients with RA. This differential treatment exposure likely neutralized circulating IL-17A levels, thereby masking intergroup differences. Furthermore, previous studies have demonstrated that IL-17A levels in the synovial fluid of patients with AS are significantly higher than those in peripheral blood, suggesting limitations of sampling sites.^[26]^

Another noteworthy result is the combination of DMARDs and biologics. Studies have revealed that in the treatment of RA, combination therapy of conventional synthetic DMARDs (e.g., methotrexate, MTX) with biologics can synergistically modulate key cytokine networks. Specifically, MTX significantly inhibits IL-6 secretion, whereas TNF inhibitors (e.g., etanercept) block TNF-α bioactivity. This dual pathway inhibition in combination therapy leads to markedly reduced levels of pro-inflammatory cytokines, including IL-6, TNF-α, and IL-17, compared with monotherapy, thereby retarding joint destruction.^[29]^ This study demonstrates that the levels of IL-1β and IL-6 in patients with RA treated with DMARDs combined with biologics were higher than those in patients treated with DMARDs alone, which may suggest the enhancement of inflammation in patients with RA treated with DMARDs combined with biologics.

Many studies have not further subdivided the drugs used by patients because the use of drugs, alone or in combination, may significantly impact the serum levels of cytokines in patients.^[30,31]^ Therefore, the effects of the drug taken by the patient on cytokine levels must be considered when assessing disease activity and monitoring drug responses.

It is important to note that this study has some limitations. First, the sample size was small and may not represent the entire RA and AS population. The study sample was derived from eastern China, where the HLA-B27 positivity rate among patients with AS was 86.2%, significantly higher than the 75% reported in Caucasian populations, which may limit the generalizability of the findings.^[32]^ Furthermore, the utilization rate of DMARDs in patients with RA was 95.72%, significantly higher than the 78% recommended by the European League Against Rheumatism guidelines for early combination therapy strategies, which may reflect variations in clinical practice.^[33]^ Future studies could consider incorporating data from different ethnic groups and healthcare institutions, thereby enhancing the generalizability of findings. Second, owing to the limited study time, we did not record changes in cytokine levels in the same patient before and after treatment with the biological agents. As a single-center retrospective observational study, this study did not allow the determination of causal relationships between treatment and cytokine changes. Future studies should adopt a prospective cohort design, which can more accurately assess such relationships through standardized baseline assessments (e.g., uniform cytokine detection methods), dynamic follow-up at predefined intervals (e.g., 3-, 6-, and 12-month time points), and endpoint event monitoring (e.g., progression of joint erosion, serious infections). Nevertheless, our findings provide valuable insights into the clinical features, laboratory markers, and cytokine profiles of patients with RA and AS. The observed differences in medication use reflect the treatment strategies used for these 2 conditions. The significant differences in cytokine levels between the RA and AS groups and after treatment with different drugs suggest that these diseases involve different inflammatory pathways and mechanisms of immune dysregulation.

## Acknowledgments

We would like to thank Professor Dong Chang, an epidemiologist, for analyzing the data results of this study.

## Author contributions

**Data curation:** Ze Zhang, Bing-He Zhou.

**Funding acquisition:** Ze Zhang, Dong Chang, Xin Dou.

**Investigation:** Liang Hu, Ming-Chao Li.

**Methodology:** Dong Chang.

**Writing – original draft:** Xin Dou.

**Writing – review & editing:** Xin Dou.

## Supplementary Material


