# Supplementary figures and images for: Relationship between biologic therapy and cytokine levels in patients with inflammatory arthritis
Source: Medicine (Baltimore). 2025 Jun 20;104(25):e42953. doi: 10.1097/MD.0000000000042953 (PMC12187313; doi:10.1097/MD.0000000000042953)

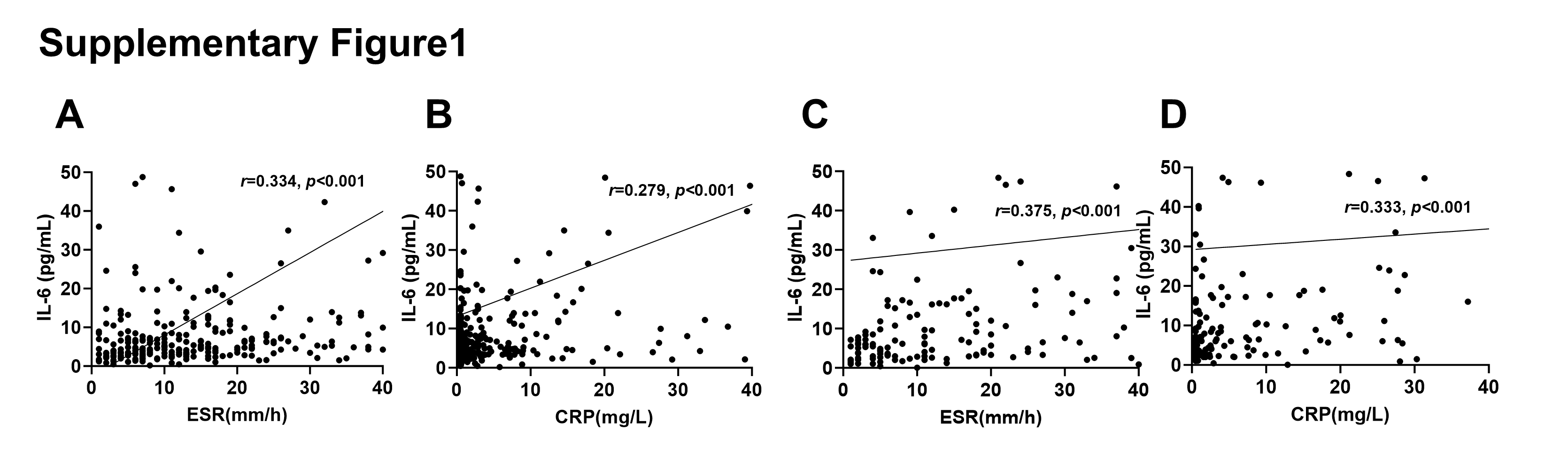

Supplement: Supplementary file 1 [file medi-104-e42953-s001.docx]
