# Supplementary material for: Relationship between biologic therapy and cytokine levels in patients with inflammatory arthritis
Source: Medicine (Baltimore). 2025 Jun 20;104(25):e42953. doi: 10.1097/MD.0000000000042953 (PMC12187313; doi:10.1097/MD.0000000000042953)
Supplement: Supplementary file 2 [file medi-104-e42953-s002.docx]

Supplementary Table 1. Effect of treatment with disease-modifying antirheumatic drugs or biologics on different cytokine levels in ankylosing spondylitis.

| Characteristics | HC | AS | | | p-value |
| --- | --- | --- | --- | --- | --- |
| DMARDs | - | - | + | + |  |
| Biologics | - | - | - | + |  |
| Number of participants | 125 | 11 | 17 | 82 |  |
| IFN-α, pg/mL | 0.75 (0.35, 1.34) | 0.87 (0.75, 1.26) | 0.75 (0.32, 1.50) | 0.86 (0.45, 1.43) | 0.559 |
| IFN-γ, pg/mL | 1.38 (0.67, 2.32) | 1.16 (0.65, 1.75) | 1.40 (0.47, 2.26) | 1.18 (0.21, 1.96) | 0.249 |
| IL-10, pg/mL | 2.11 (1.26, 2.95) | 1.02 (0.83, 2.47) | 1.19 (0.88, 1.91) | 1.57 (1.06, 2.41) | 0.004 |
| IL-12P70, pg/mL | 0.60 (0.39, 1.02) | 0.69 (0.34, 1.38) | 1.02 (0.53, 1.81) | 0.99 (0.44, 1.53) | 0.018 |
| IL-17A, pg/mL | 0.78 (0.33, 2.40) | 1.76 (0.33, 5.00) | 2.13 (0.00, 8.20) | 1.01 (0.00, 7.62) | 0.664 |
| IL-1β, pg/mL | 0.60 (0.24, 1.00) | 0.88 (0.34, 1.68) | 1.13 (0.54, 1.56) | 0.98 (0.60, 1.68) | <0.001 |
| IL-2, pg/mL | 0.92 (0.32, 1.53) | 0.95 (0.26, 1.28) | 0.94 (0.38, 1.45) | 0.92 (0.56, 1.39) | 0.989 |
| IL-4, pg/mL | 0.88 (0.47, 1.75) | 0.84 (0.58, 1.60) | 0.83 (0.20, 2.03) | 0.84 (0.21, 1.70) | 0.976 |
| IL-5, pg/mL | 0.41 (0.18, 0.68) | 0.54 (0.28, 1.05) | 0.44 (0.22, 0.76) | 0.44 (0.23, 0.69) | 0.651 |
| IL-6, pg/mL | 2.58 (1.74, 3.70) | 3.58 (2.16, 10.99) | 3.42 (1.90, 4.87) | 4.23 (2.42, 8.99) | <0.001 |
| IL-8, pg/mL | 7.14 (4.51, 10.6) | 7.71 (5.91, 11.35) | 7.46 (5.10, 8.54) | 7.16 (5.08, 9.17) | 0.759 |
| TNF-α, pg/mL | 0.97 (0.54, 1.54) | 0.82 (0.29, 2.25) | 0.85 (0.16, 2.24) | 1.01 (0.40, 1.88) | 0.942 |
